# Anti-tumor effect and hepatotoxicity mechanisms of psoralen

**DOI:** 10.3389/fphar.2024.1442700

**Published:** 2024-08-05

**Authors:** Dandan Meng, Yanling Dong, Qingxin Shang, Ziyuan Sun

**Affiliations:** ^1^ College of Traditional Chinese Medicine, Shandong University of Traditional Chinese Medicine, Jinan, Shandong, China; ^2^ Department of Breast and Thyroid Surgery, Affiliated Hospital of Shandong University of Traditional Chinese Medicine, Jinan, Shandong, China

**Keywords:** psoralen, tumor, apoptosis, hepatotoxicity, molecular mechanism, natural products

## Abstract

In recent years, natural products have gradually become an important source for new drug development due to their advantages of multi-components, multi-targets, and good safety profiles. Psoralen, a furanocoumarin compound extracted from the traditional Chinese medicine psoralea corylifolia, is widely distributed among various plants. It has attracted widespread attention in the research community due to its pharmacological activities, including antitumor, anti-inflammatory, antioxidant, and neuroprotective effects. Studies have shown that psoralen has broad spectrum anti-tumor activities, offering resistance to malignant tumors such as breast cancer, liver cancer, glioma, and osteosarcoma, making it a natural, novel potential antitumor drug. Psoralen mainly exerts its antitumor effects by inhibiting tumor cell proliferation, inducing apoptosis, inhibiting tumor cell migration, and reversing multidrug resistance, presenting a wide application prospect in the field of antitumor therapy. With the deepening research on psoralea corylifolia, its safety has attracted attention, and reports on the hepatotoxicity of psoralen have gradually increased. Therefore, this article reviews recent studies on the mechanism of antitumor effects of psoralen and focuses on the molecular mechanisms of its hepatotoxicity, providing insights for the clinical development of low-toxicity, high-efficiency antitumor drugs and the safety of clinical medication.

## 1 Introduction

Cancer, characterized by its high recurrence rate and mortality, is one of the major diseases threatening human health and life. According to the latest data published in the official journal of the American Cancer Society, there are approximately 18.1 million new cancer cases and 9.6 million cancer-related deaths globally (Sung et al., 2021). Surgery, radiotherapy, and chemotherapy are currently the main clinical treatments for malignant tumors. However, they often cause severe adverse reactions in the body and tumor cell drug resistance, leading to unsatisfactory prognosis and therapeutic effects. In recent years, the effectiveness and safety of natural products in cancer treatment have attracted widespread attention from scholars around the world. Natural products can not only intervene in the process of tumor cells but also improve the adverse reactions caused by chemotherapy, enhancing the quality of life of cancer patients. Current research on cancer treatment mainly focuses on molecular targeting, immunotherapy, and the field of natural products monomers. Studies have shown that natural products has advantages in enhancing efficacy, reducing toxicity, improving quality of life, and extending survival periods in the prevention and treatment of tumors. Many effective anti-cancer drugs have been discovered and developed from natural products (Chopra and Dhingra, 2021).

**FIGURE 1:**
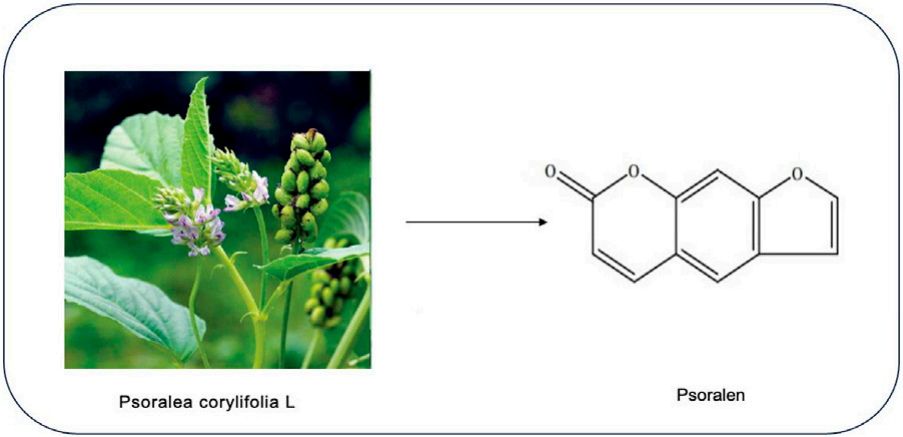
Chemical structure of PSO.

Psoralen (PSO) is a furanocoumarin compound derived from the traditional Chinese herb psoralea corylifolia L., known for its strong pharmacological activity, low toxicity, good bioavailability, and therapeutic effectiveness (Thakur et al., 2020). It is the main active ingredient of Psoralea corylifolia L., with the molecular structure of 7H-Furo[3,2-g]chromen-7-one (molecular weight: 186.16; molecular formula: C3H6O3) (Sui et al., 2020), as shown in Figure 1. PSO is widely distributed in various plants and has shown great medicinal potential, attracting widespread attention in the research community. In recent years, natural products or patent medicines containing coumarins have been widely used in clinical treatments, and studies have found they possess multiple pharmacological activities, including antitumor (Wang et al., 2018), neuroprotective (Somani et al., 2015), anti-inflammatory (Du et al., 2020), and antioxidant effects (Seo et al., 2014), suitable for treating tumors, rheumatoid arthritis, leukemia, Alzheimer’s disease, and other conditions. This suggests a broad application prospect for PSO. With the increasing attention to the safety of PSO, the studies on liver toxicity of PSO have gradually increased in recent years, and there have been reports on clinical cases of liver toxicity. Long-term or excessive use of PSO or its compound will cause damage mainly caused by abnormal liver function, and its adverse reactions limit further clinical use. Therefore, the mechanism of hepatotoxicity of PSO was discussed and studied in order to provide some reference for the attenuation compatibility of PSO and the improvement of drug safety. Meanwhile through literature review, we found that several studies have focused on its pharmacological effects, yet a comprehensive and systematic review on the molecular mechanisms of PSO’s antitumor effects has not been conducted. Therefore, this article reviews recent studies on the antitumor mechanisms of PSO and summarizes its mechanisms of hepatotoxicity, aiming to provide references for the clinical development of low-toxicity, high-efficiency antitumor drugs and the safety of clinical medication.

## 2 Antitumor molecular mechanisms of PSO

### 2.1 Induction of tumor cell apoptosis

**FIGURE 2:**
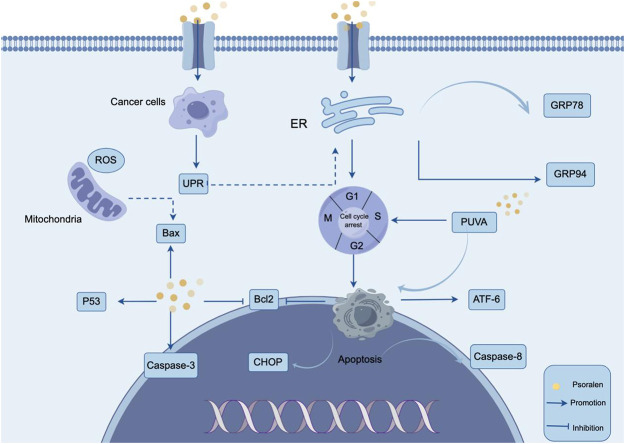
Mechanism of PSO inducing tumor cell apoptosis. PSO can lead to apoptosis of tumor cells by inducing cell cycle arrest and activating endoplasmic reticulum stress. PSO treatment resulted in elevated levels of ATF-6 and CHOP proteins, with a decrease in Bcl-2 protein levels.

Apoptosis is a form of programmed cell death, serving as a critical physiological mechanism to limit the expansion of cell populations, thereby maintaining tissue homeostasis or eliminating potentially harmful cells (Morana et al., 2022). Promoting apoptosis has become one of the important strategies in antitumor therapy (Carneiro and El-Deiry, 2020). Apoptosis can be regulated through multiple pathways, including the endoplasmic reticulum stress-mediated apoptotic signaling pathway (Oakes and Papa, 2015). Under stress conditions, the endoplasmic reticulum environment and protein maturation are disrupted, leading to the accumulation of misfolded proteins and the formation of a typical stress response, known as the unfolded protein response (UPR) (Pandey et al., 2019). UPR protects cells from stress and helps them re-establish homeostasis, while prolonged endoplasmic reticulum stress activates UPR and induces apoptosis (Adams et al., 2019; Al-Hetty et al., 2023). Li et al. through *in vitro* studies, found that PSO treatment of MG-63 and U2OS osteosarcoma cells induced cell cycle arrest at the G0/G1 phase, promoting apoptosis in MG-63 and U2OS cells (Li and Tu, 2022). Further research revealed that PSO treatment resulted in elevated levels of ATF-6 and CHOP proteins, with a decrease in Bcl-2 protein levels, suggesting that PSO-induced apoptosis is related to endoplasmic reticulum stress. In summary, PSO activated endoplasmic reticulum stress in osteosarcoma cells, further inducing apoptosis. Wang et al. further explored the mechanism of action of PSO in inhibiting liver cancer cells through *in vitro* experiments (Wang X. et al., 2019). They confirmed that PSO could induce the production of unfolded proteins and cause endoplasmic reticulum stress in SMMC7721 liver cancer cells. The results showed that PSO significantly promoted the expression of GRP78 and GRP94, inducing the unfolded protein response and leading to liver cancer cell apoptosis. Further findings indicated that PSO could cause cell cycle arrest at the G1 phase, significantly induce endoplasmic reticulum stress in a dose-dependent and time-dependent manner, leading to liver cancer cell apoptosis and inhibiting liver cancer progression. This suggests that PSO may be a new therapeutic option for the prevention and treatment of hepatocellular carcinoma in the future. caspases are a class of cysteine proteases that have long been considered a key component of the apoptosis pathway (Song T. H. et al., 2019). the tumor suppressor p53 plays a crucial role in inhibiting tumor growth and inducing apoptosis, controlling cell death through interactions with other important apoptotic molecules, including members of the bcl-2 family (Vidal and Koff, 2000; Zehir et al., 2017; Tan et al., 2019). jiang et al. found that in psoralea corylifolia-treated smmc-7721 cells, caspase-3 activity increased in a dose-dependent manner, levels of p53 and bax proteins were elevated, while the expression of bcl-2 showed a dose-dependent decrease (Jiang and Xiong, 2014). These results indicate that PSO suppresses the growth of SMMC-7721 human liver cancer cells through a mechanism that induces apoptosis by regulating the activity of caspase-3 and the expression of p53 and Bcl-2/Bax proteins. Lu et al. (2014) discovered that PSO and isopsoralen have inhibitory effects on the growth of osteosarcoma xenografts in nude mice, inducing tumor cell apoptosis or necrosis without significant toxic side effects within the therapeutic dose range. This suggests that PSO, by regulating the activity of proteins such as caspase-3 to induce cancer cell apoptosis, represents a promising anticancer drug. Studies have discovered that apoptosis in CTCL (Cutaneous T-Cell Lymphoma) cells can be induced through a model combining treatment with PSO and long-wave ultraviolet light (Photochemotherapy with PSO and ultraviolet A, PUVA). PUVA treatment leads to G2/M cycle arrest and apoptosis in MyLa and HuT-78 cells, with an increase in the expression of pro-apoptotic mitochondrial genes Bax, BAK, PUMA, and a decrease in Bcl-2 expression. This suggests that interferon-α (IFN-α) enhances PUVA-induced apoptosis in skin lymphoma cell lines through the JAK1 pathway, demonstrating the synergistic effect of IFN-α (Liszewski et al., 2017). The study indicates that mitochondrial damage may occur in lymphoblastoid cell lines following PUVA treatment, leading to apoptosis (Canton et al., 2002). Viola et al. (2007) found that two PSO derivatives, 8-methoxyPSO (8-MOP) and the coumarin derivative angelicin, significantly induce apoptosis 24 h after UVA irradiation. Under UVA exposure, these compounds induce a significant decline in mitochondrial function and activate caspase-3, caspase-8, and caspase-9 (Viola et al., 2007). Sun et al. (2013) explored the regulatory role of PUVA on apoptosis and the apoptotic signaling pathway in human leukemia NB4 cells. It was found that the apoptosis rate of NB4 cells increased in a dose- and time-dependent manner with different concentrations of PSO under the influence of ultraviolet A (UVA) for 0 and 5 min, with the highest apoptosis rate at a concentration of 40 mg/mL PSO after 5 min of UVA exposure. This indicates that PUVA can induce apoptosis in NB4 cells and activate the Caspase-3 and Caspase-8 genes *in vitro*. Glioma is the most common primary brain tumor in adults. The World Health Organization classifies it into grades I, II, III, and IV (Miller et al., 2019), with higher grades indicating greater malignancy. Approximately 100,000 people are diagnosed with diffuse gliomas worldwide each year, and the mortality rate is extremely high (Bray et al., 2018; Molinaro et al., 2019). Unfortunately, almost all high-grade gliomas will recur, and currently, there are no reported treatment methods that are both safe and low in toxicity (Cloughesy et al., 2020). Wu et al. through network pharmacology methods, discovered that the genes PIK3CA, PIK3CB, and PIK3CG are highly related to the treatment of gliomas with PSO. Further *in vitro* studies found that PSO significantly promoted the early apoptosis of glioma U87 and U251 cells, and the apoptotic capability increased with the concentration of PSO, showing a significant concentration dependence (Wu et al., 2022). This suggests that PSO has a good *in vitro* anti-glioma effect. The mechanism of PSO inducing apoptosis is shown in Figure 2.

### 2.2 Inhibition of tumor cell proliferation

**FIGURE 3:**
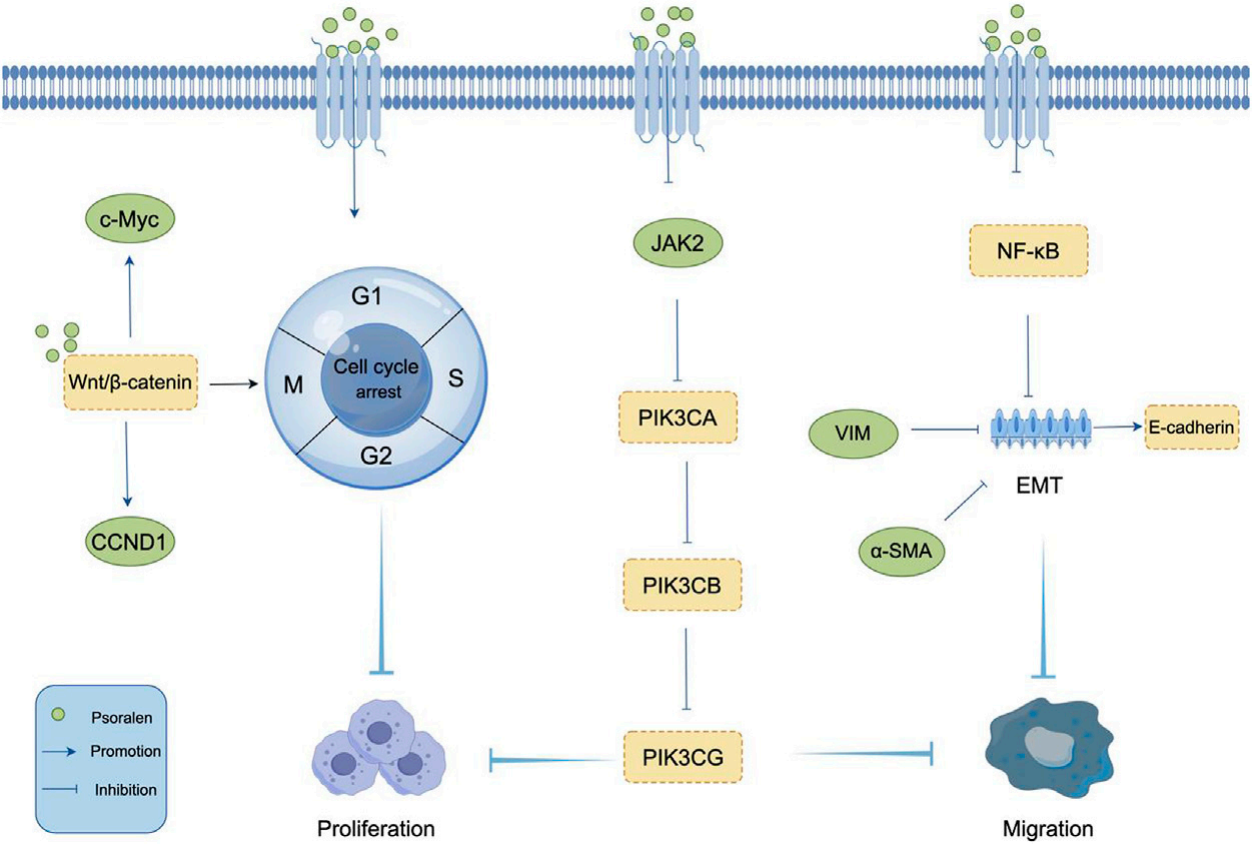
Mechanism of PSO inhibiting tumor cell Proliferation and Migration. PSO inhibits tumor cell proliferation by regulating cyclin and Wnt/β-catenin pathway. PSO prevents tumor cell migration by inhibiting EMT and upregulating the epithelial marker E-cadherin.

Proliferation, as one of the fundamental cellular functions constituting life, is a process that occurs in a strictly controlled and orderly manner (Goodlad, 2017). Unrestricted proliferation is one of the three main characteristics of cancer cells, and inhibiting tumor cell proliferation is a common method in the clinical treatment of tumors. Osteosarcoma is the most common primary bone sarcoma and a leading cause of cancer death among children and adolescents. It accounts for 3%–6% of all childhood cancers, with its incidence among the most common cancers in children and adolescents second only to lymphomas and brain cancer (Zhu et al., 2016; Simpson and Brown, 2018). Li et al. through colony formation assays, showed that the number of colonies in MG-63 and U2OS cells treated with PSO was significantly reduced compared to the control group, indicating that PSO inhibits the colony formation of osteosarcoma cells (Li and Tu, 2022). This further suggests that PSO can inhibit the proliferation of osteosarcoma cells and suppress the progression of osteosarcoma, providing a molecular basis for the further development of PSO as a novel anticancer drug for the treatment of human osteosarcoma. Additionally, a team observed the inhibitory effect of PSO on the proliferation of liver cancer SMMC7721 cells through *in vitro* experiments and further explored its relationship with endoplasmic reticulum stress (Wang X. et al., 2019). The results suggest that PSO inhibits the proliferation of SMMC7721 cells by causing cell cycle arrest at the G1 phase. Moreover, it can cause endoplasmic reticulum expansion and dysfunction, thereby continuously inducing endoplasmic reticulum stress, leading to the apoptosis of liver cancer cells. Cell cycle dysregulation is a significant characteristic of tumor cells, especially in malignant transformation, making cell cycle proteins important targets of PSO’s anticancer effects. Overexpression of these proteins can shorten the G1 phase and advance cells into the S phase, leading to continuous proliferation and increased risk of carcinogenesis (Mills et al., 2017). Wang et al. were the first to systematically and detailedly describe the anticancer effects of PSO on human breast cancer MCF-7/ADR cells (Wang et al., 2016a). They discovered that PSO could block cells in the G1 and G2 phases, significantly reducing the proportion of cells in the S phase. However, there was no significant difference in the number of apoptotic cells after treating MCF-7/ADR cells with PSO for 48 h. This indicates that PSO inhibits the proliferation of MCF-7/ADR breast cancer cells by blocking the cell cycle. However, another study reported that PSO at 10 μM and 30 μM could promote the progression of MCF-7 cells from the G1 to the S phase, suggesting that PSO’s regulatory effects on the cell cycle of different tumor cells vary (Shen et al., 2007). The classic Wnt/β-catenin pathway plays a key role in regulating tumorigenesis by blocking the cell cycle at different stages. Wang et al. (2018) findings show that PSO inhibits cell proliferation in a dose-dependent manner by inducing G0/G1 phase block in MCF-7 cells and G2/M phase block in MDA-MB-231 cells. After treatment with PSO, the expression of Wnt/β-catenin target genes (such as CCND1 and c-Myc) in MCF-7 and MDA-MB-231 cells was regulated to varying extents. This suggests that PSO can induce cell cycle arrest in MCF-7 and MDA-MB-231 cells, possibly related to its inhibition of Wnt/β-catenin transcriptional activity. The mechanism of PSO inhibiting proliferation is shown in Figure 3.

### 2.3 Inhibition of tumor cell migration

Migration and invasion are two major biological characteristics of malignant tumors. Epithelial-mesenchymal transition (EMT) is closely related to the invasion and metastasis of tumors, serving as a crucial mechanism facilitating cancer metastasis. During the progression of malignant tumors, EMT is activated, leading to epithelial cells losing cell-cell adhesion molecules like E-cadherin and acquiring mesenchymal markers such as N-cadherin, Vimentin, and α-SMA, related to cell polarity and the cytoskeleton (Dongre and Weinberg, 2019; Georgakopoulos-Soares et al., 2020; Brabletz et al., 2021; Ha et al., 2021). Therefore, targeting EMT could be an effective strategy for treating malignant and metastatic tumors. Wang et al. (2016a) determined the effect of PSO on the migration of MCF-7/ADR cells through wound healing assays, finding that PSO could inhibit the migration of MCF-7/ADR cells. They discovered that in MCF-7/ADR cells treated with PSO, the epithelial marker E-cadherin was significantly upregulated, while the mesenchymal markers Vimentin and α-SMA were significantly downregulated, suggesting that PSO could be a negative mediator of EMT and metastasis in MCF-7/ADR cells. PSO was also found to inhibit the activation of NF-κB necessary for EMT. Further research showed that PSO significantly reduced the migration capabilities of U87 and U251 glioma cells (Wu et al., 2022). *In vitro* experiments demonstrated that after 24 and 48 h of administration of 10 μM and 30 μM PSO, the migration abilities of U87 and U251 cells were significantly inhibited. It is speculated that PSO inhibits tumor cell proliferation and migration by reducing the expression of the genes JAK2, PIK3CA, PIK3CB, and PIK3CG. Breast cancer is one of the most prevalent cancers worldwide and remains the most common malignant tumor among women, leading to the highest number of cancer-related deaths among females (Nagini, 2017). Thanks to advancements in diagnostic and imaging technologies, along with early screening, the mortality rate of breast cancer has significantly decreased. However, the prognosis for breast cancer patients remains poor due to the risks of recurrence and metastasis (Chetlen et al., 2016; Li et al., 2016). Current treatments for breast cancer mainly include surgery, endocrine therapy, radiotherapy, chemotherapy, and targeted therapy (Mcdonald et al., 2016; Pan et al., 2017; Peart, 2017), but these treatments often come with severe adverse reactions (TAYLOR and KIRBY, 2015; Condorelli and Vaz-luis, 2018; Lacouture and Sibaud, 2018). In recent decades, nanoparticle-assisted combination drug therapy has made some progress in treating various types of cancer (Rawal and Patel, 2019). Polymer–lipid hybrid nanoparticles (PLNs) are a promising drug delivery system that has been widely used in the treatment of metastatic breast cancer (Zhu et al., 2015; JIang et al., 2016). Liu et al. (2021) found that PSO-loaded polymeric lipid nanoparticles (PSO-PLNs) could enhance the inhibitory effect of paclitaxel (PTX) on cell invasion and metastasis, suggesting the potential of PSO-PLNs as an adjuvant therapy against human BC cell metastasis. PTX combined with PLNs demonstrated a significant ability to inhibit cell migration and invasion activities related to the expression of IRAK1 and NF-κB in MDA-MB-231 breast cancer cells. These findings indicate that the combination of PTX and PSO-PLNs is a promising strategy for effectively treating BC metastasis. Evaluating nanoparticle formulations in animal models is also crucial for understanding their potential therapeutic impact. The mechanism of PSO inducing migration is shown in Figure 3.

### 2.4 Reversing multidrug resistance

**FIGURE 4:**
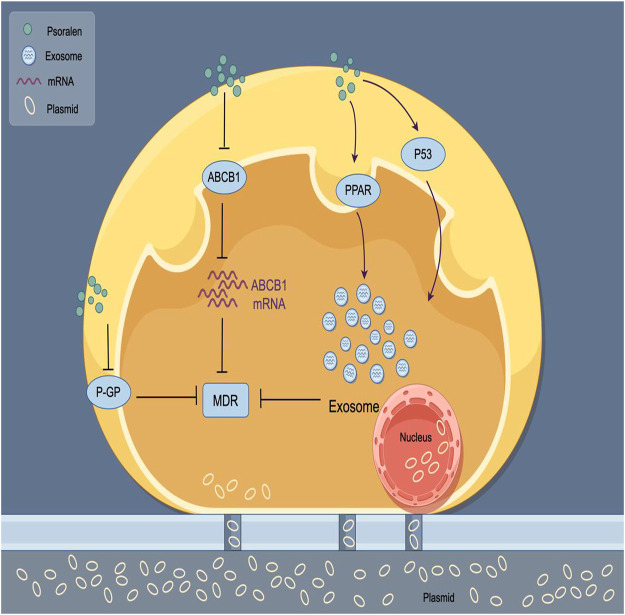
Mechanism of PSO reversing tumor cell MDR. PSO regulates the spread of exosome drug resistance through the PPAR and P53 signaling pathways, thereby inhibiting tumor cell drug resistance.

Drug resistance refers to the tolerance developed by tumor cells to anticancer drugs, which significantly diminishes the therapeutic effects of these medications. It is primarily categorized into primary resistance, secondary resistance, and multidrug resistance (MDR). Cancer poses a significant threat to global human health, and chemotherapy is one of the most common and effective strategies for treating cancer (Siegel et al., 2021). However, drug resistance and adverse reactions are widespread, constituting major obstacles and challenges in current cancer treatment (Nurgali et al., 2018). In recent years, the feasibility of using traditional Chinese medicine to combat MDR has garnered considerable attention and has begun to be explored preliminarily (Chai et al., 2010). ABCB1, a member of the ATP-binding cassette family, has been found to be associated with drug resistance and transport (Calatozzolo et al., 2005). Studies have shown that the addition of PSO made A549/D16 cells dose-dependently resensitize to the toxicity of docetaxel (DOC); further *in vitro* experiments found that PSO significantly reduced the ABCB1 mRNA levels in A549/D16 lung cancer cells (Hsieh et al., 2014). When used in combination with DOC, the levels of ABCB1 mRNA were further decreased. This suggests that PSO can inhibit the activity of the ABCB1 promoter, downregulate the expression of the ABCB1 gene, inhibit the function of ABCB1, and ultimately sensitize resistant cells to chemotherapy-induced death, thereby effectively reversing multidrug resistance. This capability positions PSO as a potential remedial measure to overcome MDR involving ABCB1. Identifying the resistance mechanisms of different chemotherapeutic drugs in various tumor cells and implementing comprehensive targeted regulation could facilitate the reversal of chemotherapy resistance. Wang et al. (2016a) explored the effect of PSO on MDR in breast cancer cells through *in vitro* experiments. They discovered that PSO at a concentration of 8 μg/mL had a reversal fold of 3.39 in MCF-7/ADR cells, indicating that PSO could significantly reverse MDR, increasing the toxicity of adriamycin (ADR) to MCF-7/ADR cells. The release of exosomes has been proven to play a key role in resistance. Targeting the transfer of exosomes from resistant cells to sensitive cells might be a method to overcome certain resistances. The PPAR signaling pathway regulates the level of ceramide synthesis, an important regulatory molecule for exosome secretion (Trajkovic et al., 2008; Wang G. et al., 2012). Research has found that PSO can reduce the spread of resistance via exosomes through the PPAR and P53 signaling pathways, thus overcoming resistance (Wang et al., 2016b). The human ABCB1 gene, also known as multidrug resistance 1 (MDR1), encodes P-glycoprotein (P-gp). The MDR1 phenotype is commonly observed in breast cancer, producing chemotherapeutic resistance (Leonard et al., 2003). Jiang et al. used an adriamycin (ADR)-resistant human breast cancer cell line, MCF-7/ADR, to investigate whether PSO could reverse MDR by regulating the function of P-gp (Jiang et al., 2016). They further discovered that at certain concentrations, PSO could significantly reduce P-gp-mediated MDR in human breast cancer MCF-7/ADR cells by inhibiting the function of P-gp, affecting the reversal of MDR. This could provide new strategies for overcoming drug resistance in breast cancer in the future. The mechanism of PSO reversing MDR is shown in Figure 4. The mechanism of antitumor action of PSO is shown in Table 1.

**TABLE 1:** The antitumor efects mechanisms of PSO.

Mechanism	Cell/tissue type	Concentration	Duration of administration	Target	Ref
Induction apoptosis	MG-63, U2OS	8, 9 μg/mL	48 h	ATF-6, CHOP, Bcl-2	ADAMS et al. (2019)
	SMMC7721	40 μM	48 h	GRP78, GRP94, CyclinD1, CyclinE1	LI and TU (2022)
	SMCC-7721	10, 30, 50, 100 ug/mL	48 h	caspase-3, p53, Bcl2, Bax	VIDAL and KOFF (2000)
	Female BALB/c nude mice	320, 1600 μg/(kg·d)	10 day	AKP	JIANG and XIONG (2014)
	NB4	0, 5, 10, 2040 μg/mL	0, 5 min	Caspase-8,Caspase-3	VIOLA et al. (2007)
	U87, U251	0, 10, 30 μM	24 h	JAK2, PIK3CA, PIK3CB, PIK3CG.	CLOUGHESY et al. (2020)
Inhibition proliferation	MG-63, U2OS	0, 10, 20, 30, 40, 50, 60, 70 μg/mL	48 h	CyclinA1, CyclinB1, CyclinD1, CDK2	ADAMS et al. (2019)
	SMMC7721	10–80 μM	24, 48,72 h	MTT	LI and TU (2022)
	MCF-7/ADR	0, 21.5, 43.0, 64.5, 86.0, 107.5 μM	48 h	G0/G1 phase arres	MILLS et al. (2017)
	MCF-7, MDA-MB-23	8, 12 μg/mL	48 h	Wnt/β-catenin, Fra-1, Axin2, cyclin D1, c-Myc	SHEN et al. (2007)
	Female BALB/c nude mice	17.5 mg/kg	Twice weekly, 28 days	β-catenin/Fra-1	SHEN et al. (2007)
Inhibition migration	U87, U251	0, 10, 30 μM	024 h, 48 h	JAK2, PIK3CA, PIK3CB, PIK3CG.	CLOUGHESY et al. (2020)
	MCF-7/ADR	43.0 μM	0, 24, 48 h	NF-KB, EMT, E-cadherin, VIM, α-SMA	MILLS et al. (2017)
Reversal of MDR	A549/D16	0, 5, 10, 20 μM	24 h	ABCB1, ABCB1 mRNA	HSIEH et al. (2014)
	MCF-7/ADR	43.0 μM	48 h	MDR1	MILLS et al. (2017)
	MCF-7, MCF-7/ADR	4, 8, 12 μg/mL	48 h	MDR1 mRNA, P-gp	JIANG et al. (2016)

## 3 Mechanisms of hepatotoxicity

The liver, being the primary organ involved in drug metabolism and a crucial detoxification organ, is susceptible to drug toxicity (Hou et al., 2016). Drugs absorbed into the bloodstream are metabolized by the liver, where their metabolites or the drugs themselves can cause direct or indirect damage to this vital organ. Despite the widespread use of traditional Chinese medicine in clinical treatment due to its natural ingredients and perceived safety, reports of liver damage related to these herbal medicines have gradually increased in recent years. As research into Psoralea corylifolia and its main active component, PSO, deepens and its range of application expands, PSO has been identified as the primary cause of FP-induced hepatotoxicity (Cheung et al., 2009; Smith and Macdonald, 2014). Toxicological studies have shown that PSO can lead to multisystem damage, including to the reproductive, immune, and nervous systems, as well as to substantial organs such as the liver and kidneys, with the liver being particularly severely affected (Wang X. et al., 2012; Xia et al., 2018). Increasing evidence suggests that the mechanisms behind PSO-induced hepatotoxicity may involve a variety of factors, including bile stasis (Wang Y. et al., 2019; Huang et al., 2021), interference with liver regeneration (Zhou et al., 2018), oxidative stress responses and mitochondrial dysfunction (Xia et al., 2018), endoplasmic reticulum stress responses (Yu et al., 2020), and abnormalities in amino acid metabolism (Zhang et al., 2018). The mechanisms of PSO induced hepatotoxicity are shown in Table 2.

**TABLE 2:** Mechanisms of PSO induced hepatotoxicity.

Mechanism	Cell/tissue type	Concentration	Duration of administration	Target	Ref.
Bile stasis	Sprague-dawley rats	60 mg/kg	1, 3, 7 days	MRP4, ABCG5 ABCG8, NTCP	YU et al. (2020)
	C57BL/6J mice	80 mg/kg	3, 7, 14 days	CYP7A1, CYP27A BSEP, OSTα HMGCR, FASN	ZHANG et al. (2018)
Delaying liver regeneration	C57 BL/6 mice	200 mg·kg∼(−1)	—	PCNA, p27, p53, p21	TAJIRI and SHIMIZU (2008)
	C57BL/6 mice	400 mg/kg, 800 mg/kg	24 h	cyclin E1, p27 mTOR	MOSEDALE and WATKINS (2017)
Endoplasmic reticulum stress	HepG2 cells	50, 100, 200, 400, 600 umol/L	6, 12, 24, 48 h	PERK-eIF2α-ATF4-CHOP, ATF6-CHOP	ZHOU et al. (2018)
Oxidative stress and mitochondrial dysfunction	HepG2 cells	300, 400, 500, 600, 700, 800 µM	24 h	Nrf2, mTOR ABL1	KIM et al. (2004)
	HepG2 cells; Male ICR mice	0–1,000 μM	12, 24, 48 h	CYP1A2, GSH MDA, SOD	WANG et al. (2021)
	zebrafish	1/10 LC1, 1/3 LC1, LC1, LC10	24, 48, 72, 96 h	T-SOD, ROS, MDA, p53, puma, apaf-1, caspase-9	SMITH and MACDONALD (2014)
Other mechanisms	ICR mice	20, 40, and 80 mg/kg	3, 7, 14, 21, 28 days	CYP2D6, CYP3A4, GST-α, GST-μ, GSH	BEHRENDS et al. (2019)
	HepG2 cells; C57BL/6 mice; ICR mice	10, 25, 50, 100, 200 μM; 20, 80, 160 mg/kg	2, 12, 24, 36, 48 h; 3, 7, 14 days	CYP1A2 AhR	ZHOU et al. (2019)

### 3.1 Bile stasis

**FIGURE 5:**
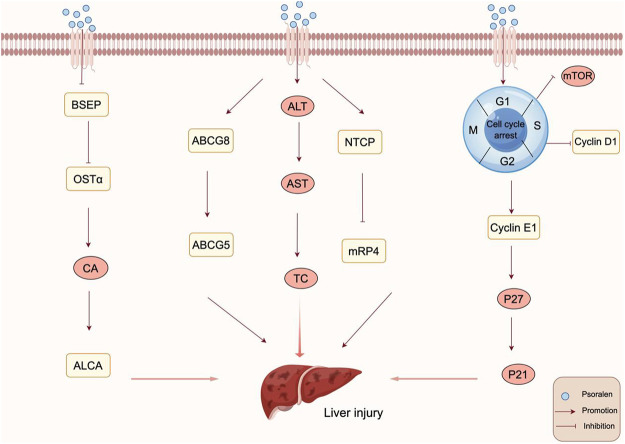
Mechanism of PSO inducing Bile Stasis and Delaying Liver Regeneration. PSO disrupt the balance of bile acid metabolism by inhibiting the expression of efflux transport proteins, thereby causing liver injury. PSO delay liver regeneration by regulating the expression of cyclin and inhibiting the mTOR signaling pathway.

Bile acids (BAs), derived from cholesterol in the liver, play a crucial role in liver function, physiology, and metabolic regulation. Bile acids have multiple functions, including preventing the formation of gallstones, promoting the excretion of cholesterol, and facilitating the absorption of lipids and nutrients in the intestines (Li and Chiang, 2009). Intrahepatic bile stasis can further lead to liver fibrosis, cirrhosis, and even liver failure. Studies have shown that approximately half of all cases of drug-induced liver injury (DILI) are associated with cholestatic dysfunction, and disruption in bile acid homeostasis is one of the mechanisms behind DILI (Tajiri and Shimizu, 2008; Mosedale and Watkins, 2017). Huang et al. explored the hepatotoxic effects of PSO on rats through *in vitro* experiments (Huang et al., 2021). They found that after administering PSO for 3 days, there was a significant upward trend in the levels of serum alanine aminotransferase (ALT), aspartate aminotransferase (AST), and total cholesterol (TC) in rats, suggesting that PSO could cause liver damage in rats after 3 days of administration. Hepatic bile acid transporters, responsible for the transport of bile acids and drugs, are crucial for preventing various cholestatic liver diseases. A decrease in or absence of the expression of hepatic bile acid transporters is a significant cause of such diseases. Further findings revealed significant changes in the mRNA and protein levels of rat hepatic bile acid transporters, with downregulation of MRP4, ABCG5, and ABCG8 proteins, and an increase in NTCP protein levels. This suggests that PSO may cause liver damage by affecting bile acid transporters, leading to disrupted transport and accumulation of bile acids within hepatocytes, which could be a potential mechanism behind PSO-induced liver injury. The transport of bile acids is coordinated by transport proteins located on the basolateral side of hepatocytes and in the bile ducts, such as the bile salt export pump (BSEP) and organic solute transporter α (OSTα). Chen et al. discovered that after administering PSO at a dose of 80 mg/kg for seven consecutive days, liver damage was induced in C57BL/6J mice (Chen et al., 2023). To further explore PSO-induced cholestatic liver injury, the bile acid content in the liver of mice was measured after 14 days of administration. It was found that PSO inhibited the expression of BSEP and OSTα, leading to impaired bile acid secretion and a significant increase in the hydrophobic bile acids CA and ALCA. This suggests that PSO may disrupt the balance of bile acid metabolism by inhibiting the expression of efflux transport proteins, thereby causing liver injury. The mechanism of PSO inducing Bile Stasis is shown in Figure 5.

### 3.2 Delaying liver regeneration

The liver is a vital organ for maintaining metabolic balance and detoxification within the body, possessing remarkable regenerative and compensatory abilities that allow it to repair and replace lost or damaged liver tissue and restore its physiological functions after injury. Liver regeneration is a complex process involving multiple cells and factors. The mammalian target of rapamycin (mTOR) acts as a sensor for various intracellular and extracellular signals, appropriately regulating biological processes including cell proliferation, metabolism, and cell cycle progression (Laplante and Sabatini, 2012). Liang et al. (2020) through studies utilizing a carbon tetrachloride (CCl4)-induced liver injury model in mice, found that PSO could induce G1/S phase arrest in hepatocytes, affecting the liver’s regenerative and repair capabilities and delaying recovery after partial hepatectomy. The preliminary mechanism may be related to the inhibition of PCNA and regulation of some cell cycle-associated protein by PSO, in which the significant upregulation of p27, p53 and p21 may play important roles. A 2/3 partial hepatectomy (PHx; also known as 2/3 PH or 70% PH) is a common *in vivo* model used to study liver regeneration. Zhou et al. (2018) through a 2/3 partial hepatectomy mouse model and *in vitro* experiments, discovered that PSO could induce mild liver injury in mice and cytotoxicity in L02 cells. The cell cycle process plays an indispensable role in tissue growth and regeneration in multicellular organisms. Further investigation into the effects of PSO on liver regeneration and cell cycle arrest *in vivo* revealed that PSO could upregulate the protein expression of cyclin E1 and p27 and downregulate cyclin D1, inducing G1/S phase arrest. This suggests that the reduced capacity for liver regeneration caused by hepatocyte cell cycle arrest may be related to the regulation of cell cycle protein expression and inhibition of the mTOR signaling pathway. The mechanism of PSO Delaying Liver Regeneration is shown in Figure 5.

### 3.3 Endoplasmic reticulum stress

The endoplasmic reticulum (ER) maintains cellular homeostasis by regulating the synthesis, folding, and modification of proteins, as well as the transport of Ca^2+^. Disruptions in ER homeostasis can affect proper protein folding, leading to endoplasmic reticulum stress (ER stress) and dysfunction. The unfolded protein response (UPR) is an evolutionary mechanism designed to restore ER homeostasis, but if ER stress is severe and unresolved, it can induce apoptosis. The UPR pathway consists of three sensor proteins: protein kinase R (PKR)-like ER kinase (PERK), inositol-requiring protein 1α (IRE1α), and activating transcription factor 6 (ATF6) (Urra et al., 2013). Numerous clinical and experimental studies have demonstrated that abnormalities in ER stress can activate apoptosis, leading to severe liver damage in clinical settings (Iracheta-Vellve et al., 2016; Ren et al., 2017). Yu et al. conducted *in vitro* experiments to explore the toxic effects of PSO on HepG2 cells. They found that PSO significantly induced liver cell death and apoptosis in a time- and dose-dependent manner (Yu et al., 2020). Moreover, PSO significantly increased the expression and transcription levels of ER stress-related markers, including Grp78, PERK, eIF2α, ATF4, and ATF6. The ER stress inhibitor 4-phenylbutyrate (4-PBA) effectively inhibited PSO-induced cell death and apoptosis, as well as the ER stress response. This suggests that PSO induces ER stress-mediated apoptosis through the PERK-eIF2α-ATF4-CHOP and ATF6-CHOP related pathways, thereby causing liver injury. Targeting ER stress presents a potential therapeutic and preventive strategy for addressing liver toxicity induced by PSO.

### 3.4 Oxidative stress and mitochondrial dysfunction

Oxidative stress is a condition characterized by an imbalance between the production of reactive oxygen species (ROS) and the body’s ability to clear them through its antioxidant defense system. It is recognized as a mechanism of chemically induced toxicity. Elevated levels of ROS can react with complex cellular molecules such as lipids, proteins, or DNA, leading to homeostatic imbalance, oxidative stress, and damage to cellular components (Cederbaum et al., 2009; Rahal et al., 2014). Studies have shown that oxidative damage can lead to liver injury when the production of ROS exceeds the antioxidant capacity of the cell (Guo et al., 2021).

**FIGURE 6:**
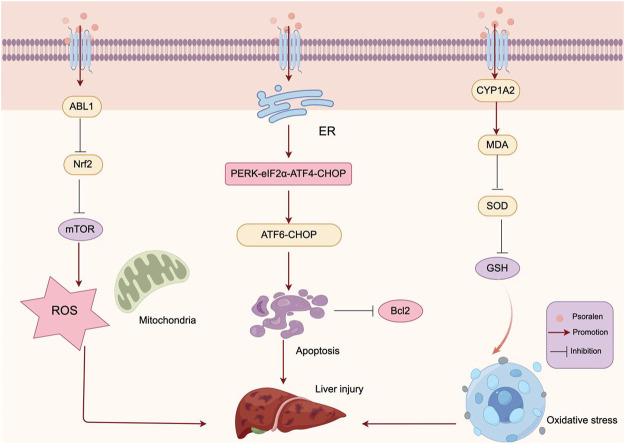
Mechanism of PSO inducing Oxidative Stress and Mitochondrial Dysfunction. PSO is metabolically activated by CYP1A2, forming reactive intermediates, thereby depleting GSH, inducing cellular oxidative stress, and liver injury. The combination of PSO and ABL1 reduced the expression of Nrf2 and mTOR, leading to elevated levels of ROS and resulting in liver injury.

The transcription factor nuclear factor erythroid 2-related factor 2 (Nrf2) plays a pivotal role in the cellular oxidative stress response, maintaining redox balance and signaling, and regulating the expression of various antioxidants (Kozieł et al., 2021). The activation of the PI3K-Akt pathway mediates positive regulatory signals of Nrf2 within the cell, participating in the antioxidative process (Kim et al., 2004). Sun et al. (2022) using proteomics and network pharmacology techniques, identified ABL1 as a direct potential target for PSO-induced hepatotoxicity and subsequently verified its toxic mechanism through *in vitro* experiments. They found that the combination of PSO and ABL1 reduced the expression of downstream Nrf2 and mTOR, leading to elevated levels of ROS and resulting in cellular damage. CYP1A2 is a major Phase I metabolic enzyme abundantly expressed in the liver (Zanger and Schwab, 2013). Research has linked PSO-induced hepatotoxicity with upregulated CYP1A2 expression (SONG L. et al., 2019). The formation of reactive metabolites catalyzed by Cytochrome P450(CYP450) and subsequent oxidative stress is a common pathway for metabolism-mediated exogenous toxicity (Jee et al., 2021; Wang et al., 2021). Zhang et al. revealed the role of CYP1A2 in PSO-induced metabolic activation and hepatotoxicity through transcriptomics and metabolomics (Zhang et al., 2023). *In vitro* and *in vivo* studies have shown that PSO-induced oxidative stress is associated with CYP1A2, where the induction of CYP1A2 leads to increased levels of MDA, and reduced activity of SOD and levels of GSH. This suggests that PSO is metabolically activated by CYP1A2, forming reactive intermediates, thereby depleting glutathione (GSH), inducing cellular oxidative stress, and hepatotoxicity. Cell survival is maintained through a balance between the levels of ROS and the cell’s antioxidative capacity (Lee et al., 2017). Xia et al. (2018) evaluated the developmental toxicity of PSO on zebrafish embryos/larvae through experiments. They discovered an increase in ROS production in the PSO treatment group; however, the activity of total superoxide dismutase (T-SOD) significantly decreased, and the levels of malondialdehyde (MDA) significantly increased. This indicates that PSO treatment induced oxidative stress during the development of zebrafish embryos, suggesting that the molecular mechanism behind PSO-induced liver damage in zebrafish might involve the generation of excessive ROS or inhibition of the organism’s antioxidative system’s clearance capacity. This triggers oxidative stress, producing a large number of free radicals and lipid peroxides, thereby causing liver damage. Moreover, mitochondrial dysfunction is also associated with the occurrence and development of hepatotoxicity (Behrends et al., 2019). Mitochondria are the main sites of energy metabolism within the cell, playing a crucial role in processes such as ATP synthesis and apoptosis. In zebrafish larvae treated with PSO, the expression levels of pro-apoptotic protein-encoding genes (p53, puma, apaf-1, caspase-9, caspase-3) were elevated, and the expression level of Bcl-2 was decreased, indicating that PSO induces apoptosis in zebrafish larvae through a mitochondria-dependent pathway (Xia et al., 2018). Additionally, gene expression analysis results suggest that PSO induces liver developmental toxicity through oxidative stress, apoptosis, and abnormalities in energy metabolism. The mechanism of PSO inducing Oxidative Stress and Mitochondrial Dysfunction is shown in Figure 6.

### 3.5 Other mechanisms

In addition to bile stasis, interference with liver regeneration, oxidative stress responses with mitochondrial dysfunction, and endoplasmic reticulum stress responses, the hepatotoxicity of PSO involves other related pathways such as drug metabolism and amino acid metabolism. The liver is the primary organ for drug metabolism, which includes phases of oxidation, reduction, hydrolysis, and conjugation. CYP450 enzymes, a class of heme-containing monooxygenases, are the main liver enzymes involved in Phase I drug metabolism through oxidation or reduction (Zanger and Schwab, 2013). Research by Jiang et al. found that the accumulation of PSO in the liver is a major cause of liver injury (Jiang et al., 2022). Further investigation revealed that PSO can directly bind to CYP2D6, CYP3A4, GST-α, and GST-μ, inhibiting their activity, leading to the depletion of GSH in the body, causing liver damage and resulting in disorders of drug metabolism. The combined use of PSO with glutathione can alleviate the elevation of transaminase levels caused by PSO and improve liver pathological changes, suggesting that the combination of GSH and PSO is an effective method to avoid liver damage in clinical practice. The aryl hydrocarbon receptor (AhR) is a ligand-dependent transcription factor that initiates the transcription of target genes by forming heterodimers with AhR nuclear translocator, regulating the expression of drug-metabolizing enzymes such as CYP1A and 1B (Vogel et al., 2020; Xu et al., 2021; Zhang W. et al., 2022). PSO-induced hepatotoxicity has been reported to be associated with the exogenous metabolism of cytochrome P450 (CYPs), with CYP1A2 being a significant metabolic enzyme involved in PSO-induced hepatotoxicity (Song L. et al., 2019). CYP1A2 accounts for approximately 4%–16% of the total CYP pool in the liver and is involved in the metabolism of the main components in PF (Zhou et al., 2019; Kwon et al., 2021). Zhang C. et al. (2022) discovered through *in vitro* and *in vivo* experiments that the hepatotoxicity induced by PSO in HepG2 390 cells and mice is related to the induction of CYP1A2 expression. The potential mechanism may involve the activation of AhR by these molecules, initiating the transcription of the target gene CYP1A2, leading to increased levels of CYP1A2. This is accompanied by elevated levels of CYP1A2 mRNA and protein, and significant inhibition of CYP1A2 activity *in vitro*. Metabolomics, a relatively new research technique, has become a viable tool for studying the biochemical actions of many toxic substances (Han et al., 2016). With the continuous development of metabolomics, untargeted metabolomics has been widely applied in drug safety evaluation and toxicity prediction, providing valuable information for drug-induced cardiotoxicity, hepatotoxicity, and nephrotoxicity. Research teams have observed that long-term exposure to low levels of PSO and isoPSO induces hepatotoxicity in female rats and changes in the serum metabolome. These changes include disruptions in the metabolic pathways of alanine metabolism, glutamate metabolism, the urea cycle, the glucose-alanine cycle, the ammonia cycle, and the metabolism of glycine and serine. This suggests that PSO may inhibit the synthesis of proteins from free amino acids while also preventing their use as substrates for gluconeogenesis in rats (Yu et al., 2019). Zhang et al. (2018) utilizing 1H-NMR metabolomics technology combined with conventional serum biochemistry, investigated the mechanism of liver injury induced by PSO and found that the liver is a direct target of PSO toxicity. Multivariate analysis identified seven metabolites in serum samples and fifteen metabolites in liver samples as potential biomarkers for liver injury caused by PSO. Further findings suggested that PSO might cause liver damage by disrupting the biosynthesis of valine, leucine, and isoleucine in the serum and liver. These findings provide a reference for the medicinal safety and potential risks of PSO.

## 4 Discussion

The global incidence and mortality rates of cancer continue to climb, posing a significant impact on human health worldwide. The mechanisms underlying malignant tumors are incredibly complex, making the exploration of their pathogenesis and the search for safe and effective anti-tumor drugs especially important. With the rapid advancements in medical science and the continuous refinement of bioinformatics, effective targets and pathways for natural drugs to regulate tumors are gradually being identified, and an increasing number of active monomers from traditional Chinese medicines are being discovered. PSO, a major active component of the traditional Chinese medicine Psoralea corylifolia, can inhibit tumor growth through multiple targets and has relatively good safety. Therefore, researching and developing the anti-tumor activity and mechanisms of PSO hold significant importance and have broad prospects in treating malignant tumors. PSO is widely distributed in various natural plants and has attracted widespread attention from the research community due to its good medicinal potential. It exhibits multiple pharmacological activities, including anti-tumor, neuroprotective, anti-inflammatory, and antioxidant effects, playing an important role in the treatment of various diseases. PSO’s anti-tumor effect is extensive; it not only inhibits the growth of solid tumors and primary tumor cells but also controls the invasive activity of tumor cells to prevent metastasis. PSO exerts its anti-tumor effects mainly by inducing apoptosis, inhibiting cell proliferation and migration, and reversing multi-drug resistance of tumor cells, making it a promising anti-tumor drug. With the continuous development and improvement of experimental techniques and bioinformatics, the anti-tumor effect of PSO has been fully confirmed in experiments, and its pharmacological mechanism has been elucidated at multiple levels. However, current research is mostly limited to *in vitro* experiments, lacking necessary clinical studies. Further research on its precise mechanisms, target actions, and clinical applications is needed. Besides the most common anti-tumor active components of Psoralea corylifolia, PSO and isoPSO, research on the anti-tumor effects of other chemical components is scarce. It would be beneficial to increase the research on the synthesis of derivatives of PSO and isoPSO and their anti-tumor activities to discover compounds with less toxicity and resistance. Additionally, comparing and analyzing the chemical structures of anti-tumor active components in Psoralea corylifolia, modifying and optimizing the compounds, and focusing on *in vivo* anti-tumor activity and drug processes in the body will help develop more clinically meaningful anti-tumor drugs.

The percentage of a medicinal product absorbed unmodified in the systemic circulation represent the bioavailability of that product (Pandareesh et al., 2015; Salehi et al., 2020). The bioavailability represents the actual effective concentration of these compounds at the site of drug action following their absorption from the gastrointestinal tract after oral administration of a dosage form (solution, suspension, tablet, etc.) (Chow, 2014; Salehi et al., 2018). PSO is a natural compound that is orally bioavailable. New drug forms such as nanoencapsulation have been developed to improve the bioavailability of PSO (Chen et al., 2017; Zhang et al., 2019). Yen et al. found that nanoencapsulation of PSO (viachitosan and Eudragit S100) improved the efficiency of oral drug delivery. The pharmacokinetic analysis of PSO and PSO-nanoencapsulate revealed that the bioavailability of nano-microencapsulated microspheres was 339.02% higher compared to simple microsphere suspension, indicating that nano-microcapsules exhibited superior potential in enhancing the oral absorption of PSO when compared to traditional suspensions (Yin et al., 2016). These results suggest that PSO pharmaceutical nanoformulations containing chitosan and EudragitS100 compounds have great potential for improving PSO bioavailability.

To address psoralen-induced hepatotoxicity, the dosage of psoralen can be reduced to mitigate toxicity, especially for patients with impaired liver function. Additionally, developing psoralen analogs with lower toxicity but retained efficacy is another approach. Combining psoralen with other drugs that enhance its therapeutic effects can also reduce its toxicity. Structurally modifying psoralen to enhance its therapeutic properties and reduce toxicity has been achieved by synthesizing several derivatives and analogs, such as 8-methoxypsoralen (8-MOP) and 5-methoxypsoralen (5-MOP). These modifications aim to improve psoralen’s photoreactivity and reduce side effects. Psoralen targeted delivery systems utilize nanoparticle-based systems to direct psoralen to tumor cells, minimizing exposure to healthy tissues. Researchers have developed various targeted delivery systems to improve psoralen’s therapeutic index. Encapsulating psoralen in nanoparticles can enhance its delivery to tumor cells while reducing systemic toxicity. Nanoparticles can be designed to release psoralen in response to specific stimuli in the tumor microenvironment, such as pH or temperature changes. Regarding the clinical application of psoralen, a review of relevant literature indicates that psoralen combined with UVA light (PUVA therapy) has been clinically used to treat various skin diseases, including psoriasis, vitiligo, and cutaneous T-cell lymphoma. The use of traditional Chinese medicine can lead to certain liver injuries (Herb-induced liver injury, HILI). The development of HILI is insidious, closely related to the drug’s toxicity, dosage, duration of use, and individual differences. As reports of liver toxicity caused by Psoralea corylifolia and its compound preparations increase, the material basis of Psoralea corylifolia liver toxicity has received widespread attention from scholars. PSO, as the main active component of Psoralea corylifolia, has been proven to have hepatotoxicity, and long-term use can lead to liver damage. PSO can cause liver toxicity through various pathways, such as inhibiting liver regeneration, bile stasis, oxidative stress, and mitochondrial dysfunction, causing damage to the liver. Further analysis reveals the complexity of the mechanisms behind herbal medicine-induced liver toxicity due to the diversity of toxic substances, the complex chemical components of natural drugs, and their multi-level, multi-target, and multi-pathway effects on the body, further hindering the systematic characterization and elucidation of the mechanisms of herbal liver toxicity. On one hand, the emergence of multi-omics technologies provides a powerful tool for the study of traditional Chinese medicine toxicology, describing cellular life activities at multiple levels. Its advantages lie in the specific changes in biological pathways during the onset and treatment of diseases, aligning with the characteristics of natural drugs’ multi-pathway, multi-component, and multi-target effects, to some extent solving issues of unclear action targets and undefined toxicity mechanisms in natural drug toxicity research. Therefore, it is necessary to further apply metabolomics technology to the study of traditional Chinese medicine toxicology, providing key support for rational and safe medication. On the other hand, as a natural drug with potential toxicity, a rational evaluation of the hepatotoxicity of PSO can help improve the safety and rationality of clinical medication. Establishing scientific and rational traditional Chinese medicine safety evaluation methods, further exploring the mechanisms of PSO’s toxicity reduction and the “toxicity-effect” transformation relationship, will aid in rationally evaluating the benefits and risks of using PSO, providing references for its clinical safe use, new drug development, and drug evaluation containing Psoralea corylifolia. In summary, the advantages of natural drugs in terms of safety and efficacy have attracted widespread attention from scholars. The development and utilization of traditional Chinese medicine have become a hot topic. An increasing number of natural drugs are being developed, and in the future, traditional Chinese medicine monomers are expected to be developed into new, safe, and effective anti-tumor drugs. Meanwhile, we should also pay attention to the potential liver damage caused by natural drugs, rationally evaluate their hepatotoxicity while developing and utilizing them, and maximize the advantages of natural drugs for clinical safe use, new drug research and development, and drug evaluation.
